# Differential Expression of Adenine Nucleotide Converting Enzymes in Mitochondrial Intermembrane Space: A Potential Role of Adenylate Kinase Isozyme 2 in Neutrophil Differentiation

**DOI:** 10.1371/journal.pone.0089916

**Published:** 2014-02-25

**Authors:** Ayako Tanimura, Taigo Horiguchi, Keiko Miyoshi, Hiroko Hagita, Takafumi Noma

**Affiliations:** Department of Molecular Biology, Institute of Health Biosciences, the University of Tokushima Graduate School, Tokushima, Japan; University of Iowa, United States of America

## Abstract

Adenine nucleotide dynamics in the mitochondrial intermembrane space (IMS) play a key role in oxidative phosphorylation. In a previous study, *Drosophila adenylate kinase isozyme 2* (*Dak2*) knockout was reported to cause developmental lethality at the larval stage in *Drosophila melanogaster*. In addition, two other studies reported that *AK2* is a responsible gene for reticular dysgenesis (RD), a human disease that is characterized by severe combined immunodeficiency and deafness. Therefore, mitochondrial AK2 may play an important role in hematopoietic differentiation and ontogenesis. Three additional adenine nucleotide metabolizing enzymes, including mitochondrial creatine kinases (CKMT1 and CKMT2) and nucleoside diphosphate kinase isoform D (NDPK-D), have been found in IMS. Although these kinases generate ADP for ATP synthesis, their involvement in RD remains unclear and still an open question. In this study, mRNA and protein expressions of these mitochondrial kinases were firstly examined in mouse ES cells, day 8 embryos, and 7-week-old adult mice. It was found that their expressions are spatiotemporally regulated, and Ak2 is exclusively expressed in bone marrow, which is a major hematopoietic tissue in adults. In subsequent experiments, we identified increased expression of both AK2 and CKMT1 during macrophage differentiation and exclusive production of AK2 during neutrophil differentiation using HL-60 cells as an *in vitro* model of hematopoietic differentiation. Furthermore, *AK2* knockdown specifically inhibited neutrophil differentiation without affecting macrophage differentiation. These data suggest that AK2 is indispensable for neutrophil differentiation and indicate a possible causative link between AK2 deficiency and neutropenia in RD.

## Introduction

Adenine nucleotides such as ATP, ADP, and AMP are involved in several cellular functions, including intracellular and extracellular signaling, energy metabolism, cell growth, and differentiation [1], [2]. Among these processes, energy metabolism is critical for cell viability and homeostasis. In mammalian systems, cells produce ATP through cytosolic glycolysis and mitochondrial oxidative phosphorylation depending on O_2_ availability. In somatic cells, ATP is efficiently produced by coupling respiratory chain and ATP synthase under aerobic condition [3]. Although 36 moles of ATP are produced from 1 mole of glucose in mitochondria, only 2 moles are produced in cytosol through glycolysis. Therefore, mitochondrial activity is a great advantage for differentiated somatic cell functions and homeostasis. High-energy phosphoryl transfer between ATP-generating and ATP-consuming sites is primarily mediated by creatine kinases (CK; EC 2.7.3.2) and adenylate kinases (AK; EC 2.7.4.3) [1], [2], [4], [5]. During active mitochondrial respiration, ADP transfer into the mitochondrial matrix is mediated by the adenine nucleotide translocator (ANT), which is located in IMS [6]. ADP in IMS is then rapidly exchanged with ATP by mitochondrial CK and/or AK. Thereby, CK and AK systems contribute to coordinated energy transfer and feedback signal transduction networks [7], ensuring rapid recycling of ADP for efficient mitochondrial ATP production [8], [9].

The AK family comprises nine isozymes that are located in various subcellular compartments and are distributed in tissues as follows: AK1, 5, 7, and 8 are located in the cytosol, AK2, 3, and 4 are located in the mitochondria, and AK6 is located in the nucleus [1], [2], [10]–[12]. In addition, a recent study using GFP-fusion proteins demonstrated AK9 in both cytosolic and nuclear compartments [13]. Cytosolic and organellar AK isozymes maintain adenine nucleotide homeostasis via the reaction; Mg^2+^-ATP (or GTP) + AMP ↔ Mg^2+^-ADP (or GDP) + ADP. Although AK3 and 4 are located in the mitochondrial matrix, AK2 is uniquely located in IMS, particularly in liver and kidney tissues [10], [14]. As a member of the family of ATP-AMP phosphotransferases, AK2 catalyzes the reversible transfer of a phosphoryl group between ATP + AMP and 2 ADP [1], [10]. Other kinases in IMS include creatine kinases CKMT1 and CKMT2 and nucleoside diphosphate kinase D (NDPK-D; EC 2.7.4.6). CKMT1 is expressed in most tissues, except muscle and liver, whereas sarcomeric CKMT2 is located primarily in muscle and heart tissues. Both CKMT1 and CKMT2 catalyze the reversible reaction ATP + creatine ↔ ADP + phosphocreatine [5], [15], whereas NDPK-D (also known as NME-4, NM23-H4) catalyzes the reaction ATP + GDP ↔ ADP + GTP [16].

In our previous study, we reported developmental failure in *Dak2* knockout *Drosophila melanogaster*, showing that *Dak2* knockout is lethal prior to the third larval stage [17]. Additionally, *ak2* knockdown of lepidopteran insects *Helicoverpa armigera* has reported larval growth impairment and reduction of haemocytes [18]. Furthermore, two groups independently reported that *AK2* is a responsible gene for RD [19], [20], and soon after another group reported that *Ak2* knockdown caused impaired cellular differentiation in both adipose cells and B cells [21]. In addition, recent our study demonstrated that any germ-layer specific knockdown of *Dak2* gene by RNAi treatment resulted in larval lethality, showing that ak2 is definitively required for larval developmental process of *D. melanogaster* [22]. RD is a type of severe combined immunodeficiency caused by impairment of lymphoid and neutrophil lineage development but does not involve erythroid, platelet, or macrophage lineages. Based on these findings, we hypothesized that AK2 deficiency may inhibit neutrophil differentiation through impaired ADP recycle across the mitochondrial innermembrane, and it may lead to both dysfunction of mitochondrial energy metabolism and impairment of lymphocytic and granulocytic cellular differentiation, as observed in patients with RD.

To test these hypotheses, we investigated the expression of mitochondrial kinases. Then, we analyzed the relationship between their expressions and myelocytic differentiation using hematopoietic HL-60 cells [23], [24].

## Materials and Methods

### Cells culture and reagents

Mouse embryonic stem cells, B6J-S1^UTR^, and HL-60 human promyelocytic leukemia cells were provided by the RIKEN Bio Resource Center through the National Bio-Resource Project of the Ministry of Education, Culture, Sports, Science and Technology, Japan [25], [26]. ES cells were cultured in an ES medium on mitomycin C-treated MEF feeder cells (MEF-MMC; Repro CELLS, Yokohama, Japan). ES medium comprised high-glucose DMEM containing 20% KSR (Gibco, Life technologies, Carlsbad, CA), 0.1 mM NEAA (Gibco), 1000 U/ml mouse LIF (Chemicon International, Inc., Temecula, CA), and 0.1 mM 2-ME (Sigma, St. Louis, MO). ES cells were harvested after removing MEF-MMC cells by trypsinization. HL-60 cells were maintained in a RPMI-1640 medium with 10% fetal bovine serum (FBS).

### Animals

All animal experiments were approved by the Ethics Committee for Animal Experiments of the University of Tokushima (No. 11115). In addition, experiments were performed according to the guidelines and principles for the care and use of animals at the University of Tokushima. All surgery was performed under ether anesthesia, and all efforts were made to minimize suffering. Seven-week-old male and pregnant ICR mice were purchased from Japan SLC (Shizuoka, Japan). Brain, heart, lung, stomach, large intestine, liver, kidney, thymus, spleen, bone marrow, muscle, and testis tissues were collected from anesthetized 7-week-old mice. At day 8 of gestation, pregnant mice were anesthetized, and embryo propers were carefully removed from extra-embryonic portion, such as yolk sac following laparotomy. Then, we used only embryo proper that will become a mouse body, not extra-embryonic portion for the analyses. Hereafter, embryo propers are referred to as “embryos”. ES cells, embryos, and tissues were homogenized in a lysis buffer containing 60-mM Tris-HCl, (pH 7.5), 150 mM NaCl, 5 mM EDTA, and 0.2% TritonX-100 and were then sonicated. After centrifuging at 5000 rpm for 5 min at 4°C, supernatants were collected for experiments.

### RT-PCR

Total RNA was extracted from mouse ES cells, embryos, and adult tissues using the TRI Reagent (Molecular Research Center, Cincinnati, OH) according to the product manual. After DNase I treatment, cDNA was synthesized from 1 µg of total RNA using the TaKaRa DNA PCR Kit (AMV) (TaKaRa, Kyoto, Japan). PCR reactions were performed using Go Taq Flexi DNA polymerase (Promega, Madison, WI) and following specific primer pairs: *Ak2* forward 5′-CCCAAACTGGCTGAAAAC-3′, reverse 5′-TCGCAAACACGATGTCAG-3′; *Ckmt1* forward 5′-TTCTCCCGTCTGCTGTCTG-3′, reverse 5′-TGGACAGGTCAAGATGTAGCC-3′; *Ckmt2* forward 5′-CAAGAAGAAGGATGGCCAGT-3′, reverse 5′-TTCATCACCCTAGGGTCATA-3′; *Ndpk-d* forward 5′- ATGGCTCTCAGAGTCCTTCTGTTAA-3′, reverse 5′-CATCAAAGAGAACAAGGTTTTGGAC-3′; *18S rRNA* forward 5′-TACCTGGTTGATCCTGCCAGTAGGAT-3′, reverse 5′-CCCGTCGGCATGTATTAGCTCTAGAA-3′. Following agarose gel electrophoresis, PCR products were quantitated using densitometry with a Bio-Rad ChemiDoc XRS system (Bio-Rad, Hercules, CA).

### Western blot

Western blot analyses were performed as previously described [27]. Briefly, 20 µg of total protein were loaded into 10% or 12.5% SDS-polyacrylamide gels, and electrophoresis was performed. After transfer of proteins to PVDF membranes and nonspecific epitope blocking, the following antibodies were used for immunodetection; anti-AK2 antibody [28], anti-AK2 (H-65) (Santa Cruz, Santa Cruz, CA), anti-uMtCK (N-15) (Santa Cruz), anti-CKMT2 (Abcam, Cambridge, MA), anti-nm23-H4 (H-53) (Santa Cruz), anti-GAPDH (14C10) (Cell signaling, Danvers, MA), monoclonal anti-β-actin Clone AC-15 (Sigma), human integrin alpha M/CD11b (238439) (R & D systems, Minneapolis, MN), Pan-Actin (Cell signaling), antigoat HRP IgG (Dako, Glostrup, Denmark), ECL antirabbit IgG horseradish peroxidase-linked whole antibody, and antimouse IgG (GE Healthcare, Munich, Germany). Signals were detected using the Immobilon Western Chemiluminescent HRP Substrate (Millipore, Billerica, MA) and Fuji medical X-ray film (Fujifilm, Tokyo, Japan).

### Enzyme assay

Enzyme assays were performed according to our previous report [14]. Briefly, AK1 and AK2 activities were assayed in the reaction: ATP + AMP ↔ 2 ADP. ADP formation was coupled with pyruvate kinase and lactate dehydrogenase reactions, leading to NADH oxidation. Subsequently, AK reaction rates were determined by measuring the decrease in NADH absorbance at 340 nm at 25°C. One unit of AK activity was defined as that required to produce 1 µmol of ADP per min at 25°C. AK2 activity was defined as total AK activity remaining after N-ethylmaleimide (AK1 inhibitor) treatment [29], and AK1 activity was determined by subtracting AK2 activity from total AK activity.

### Macrophage- and neutrophil differentiation from HL-60 cells

Neutrophil differentiation was induced by treating HL-60 cells (2.5×10^5^ cells/ml) with 10 µM all-trans retinoic acid (ATRA; Sigma) [23]. Macrophage differentiation was induced by treating HL-60 cells (1×10^5^ cells/ml) with 200 ng/ml phorbol myristate acetate (PMA; Sigma) [24].

### Cytological staining

Macrophage- and neutrophil-differentiated HL-60 cells were analyzed by staining with Giemsa and Wright-Giemsa solution (Muto Pure Chemicals, Tokyo, Japan), respectively, according to the manufacturer's protocol. Briefly, cells were dried for 1–3 min and were fixed in methanol for 30 s. Subsequently, macrophages were stained with Giemsa solution for 10 min and were then washed in water. Further, neutrophils were stained with Wright-Giemsa solution for 2 min. After diluting M/150 phosphate buffer, cells were stained for 8 min and were then washed.

### *AK2* knockdown

*AK2* knockdown was achieved by transfection using Silencer Select validated *AK2* siRNA (S1211, Ambion, Life technologies, Carlsbad, CA) and a Nucleofector II Cell Line Nucleofector Kit V (Lonza Japan, Tokyo). Before nucleofection, cells were seeded at 1×10^5^ cells/ml. After 3 days, 2×10^6^ cells were collected and nucleofected using program T-019 with 100 µl nucleofector solution and 6 or 9 µg *AK2* siRNA, or 9 µg of Silencer Select Negative Control #1 (Ambion) according to manufacturer's instructions. For differentiation experiments, cells were treated with differentiation-inducing reagents (ATRA and PMA) for 2 days after nucleofection.

### NBT assay

Differentiated cells (1.2×10^6^ cells) were incubated with NBT solution (Muto Pure Chemicals, Tokyo, Japan) as previously described [23], [25], [30]. Then, the cells were suspended in PBS, and NBT-positive cells (more than 200 cells) were counted and calculated the ratio of NBT positive cells.

### ROS measurement

ROS levels in *AK2*-knockdown HL-60 cells were measured during differentiation by CellROX Green Oxidative Stress Reagents (Life Technologies) according to the manufacturer's instruction. Briefly, the differentiated cells at appropriate time points were incubated with 10 µM CellROX reagent for 30min at 37°C. The cells were washed twice with PBS and measured fluorescent intensity using Varioskan Flash microplate reader (Thermo Scientific, MA, USA).

### Statistics

Each analysis was performed more than three independent materials and experiments conducted under the same experimental conditions. The image data of RT-PCR and WB were quantified using Image J software (http://rsb.info.nih.gov/ij/). The data was normalized against 18S rRNA, Pan-Actin, or protein staining density by Coomassie Brilliant Blue or Ponceau S, and calculated the means ± S.E., respectively. Student's *t*-test was performed with Microsoft Excel.

## Results

### Expression of mitochondrial kinases in adult mouse tissues

**Figure 1:**
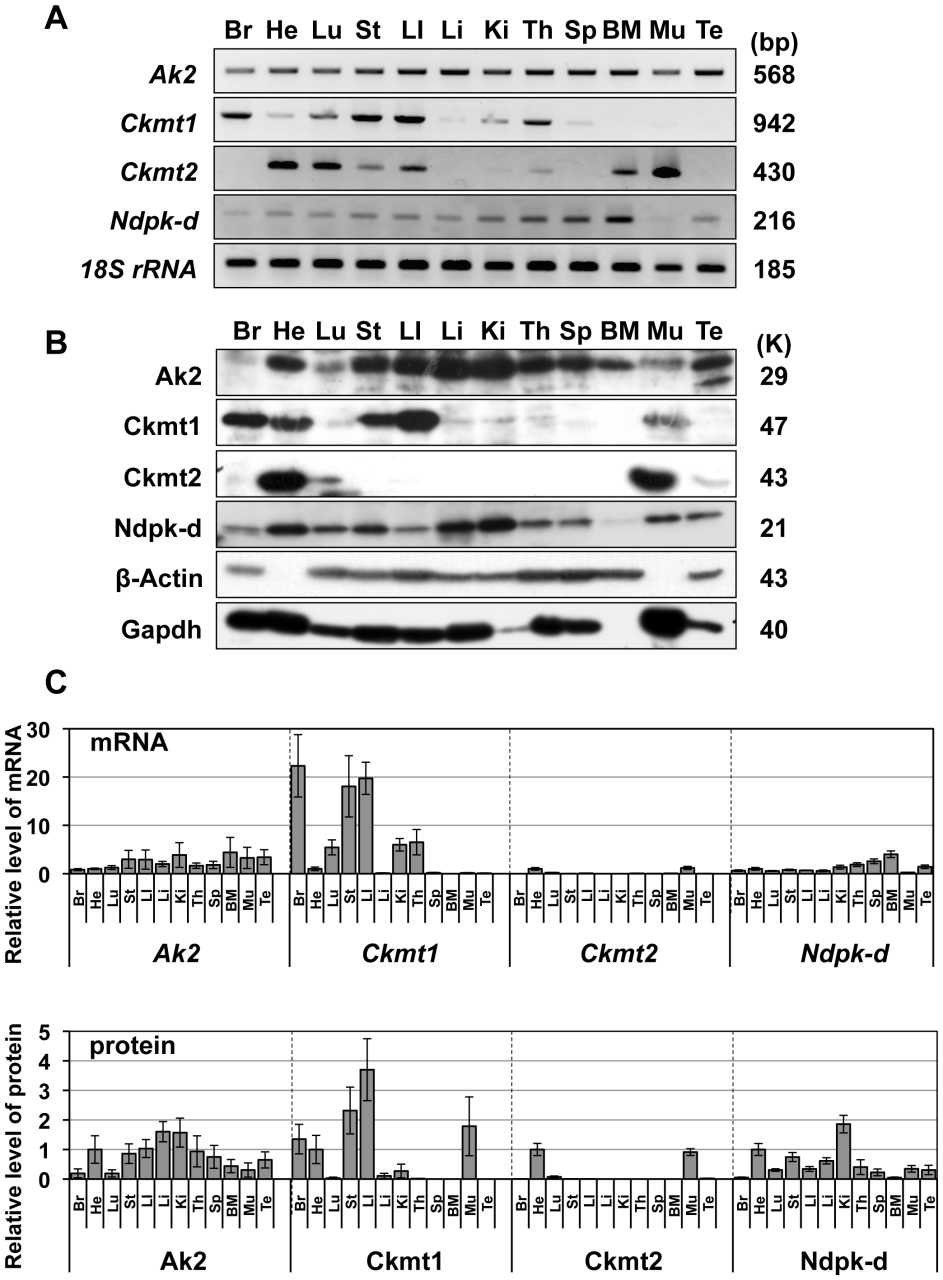
Expression of mitochondrial kinases in 7-week-old ICR mouse tissues. (A) Tissue-specific expression of *Ak2*, *Ckmt1*, *Ckmt2*, and *Ndpk-d* mRNA in adult mouse tissues. Br, brain; He, Heart; Lu, lung; St, stomach; LI, large intestine; Li, liver; Ki, kidney; Th, thymus; Sp, spleen; BM, bone marrow. Mu, skeletal muscle; Te, testis; *18S rRNA* is presented as a loading control. Sizes of PCR products are presented on the right side of the panel. (B) Tissue-specific expression of Ak2, Ckmt1, Ckmt2, and Ndpk-d proteins in adult mouse tissues. β-Actin and Gapdh are presented as loading controls. Molecular weight is shown on the right side of the panel. (C) Relative levels of mRNA and protein expression of each enzyme in adult mouse tissues. The relative level is shown compared to the value of heart as 1. Sample numbers are shown as follows; N = 4 for *Ak2* and *Ndpk-d*, N = 5 for *Ckmt1*, *Ckmt2*, Ak2, Ckmt1, Ckmt2 and Ndpk-d.

Initially, we investigated expressions of *Ak2*, *Ckmt1*, *Ckmt2*, and *Ndpk-d* mRNA in adult mouse tissues. As presented in Figure 1A, *Ak2* mRNA was detected in all 12 mouse tissues, including brain, heart, lung, stomach, large intestine, liver, kidney, thymus, spleen, bone marrow, skeletal muscle, and testis. We next examined mRNA expression of each *Ak2* isoform since two *Ak2* isoforms, *Ak2A* and *Ak2B*, have been previously reported (NM_001033966.4 and NM_016895.4) [31], [32] (Figure S1A). In adult mouse tissues, we found differential expression pattern of *Ak2A* and *Ak2B*, indicating that expression of *Ak2* isoforms could be regulated by alternative splicing in a tissue-specific manner (Figure S1B).

On the other hand, *Ckmt1* mRNA expression was detected in brain, lung, stomach, large intestine, and thymus, and not in bone marrow and testis. In addition, *Ckmt2* mRNA was highly expressed in heart and skeletal muscle, whereas *Ndpk-d* mRNA was ubiquitously expressed, although at low levels in brain and skeletal muscle.

Western blot analyses detected strong Ak2 signals in most tissues, except brain, lung, and skeletal muscle, with strongest signals in liver and kidney tissues (Figure 1B). Ckmt1 was strongly detected in brain, heart, stomach, and large intestine, but not in spleen, bone marrow, and testis. Strong Ckmt2 signals were detected in both heart and skeletal muscle. Ndpk-d was detected in almost all tissues but at very low levels in bone marrow. Figure 1C shows the relative levels of mRNA and protein expression. Ak2 protein expression was roughly correlated with mRNA expression in each tissue, whereas mRNA and protein expression of Ckmt1 and Ckmt2 were varied among tissues, suggesting that the expression of CK isozymes is tissue-specifically and post-transcriptionally regulated. In bone marrow, mRNA and proteins of Ckmt1 and Ckmt2 were not detected, and Ndpk-d expression was also very low, suggesting that Ak2 may play an exclusive role in adenine nucleotide metabolism of bone marrow.

### Expression of mitochondrial kinases in mouse ES cells and E8 embryos

**Figure 2:**
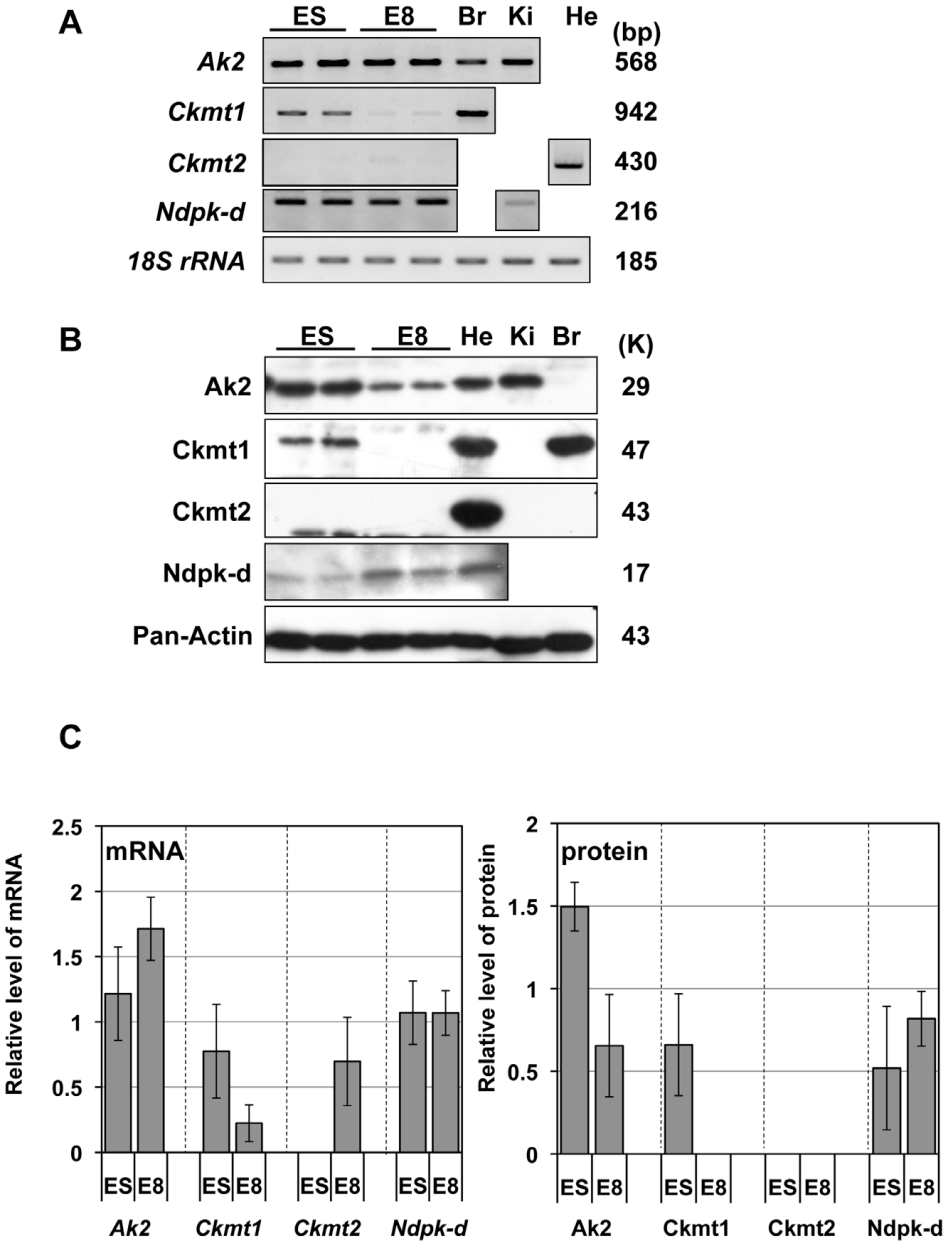
Expression of mitochondrial kinases in mouse ES cells and embryos. (A) RT-PCR analyses were performed on mouse ES cells and E8 embryos. ES, mouse ES cells; E8, mouse E8 embryos. Br, brain; Ki, kidney; and He, heart tissues were used as PCR controls. *18S rRNA* is presented as a loading control. Sizes of PCR products are presented on the right side of the panel. (B) Western blot analysis was performed on mouse ES cells and E8 embryos. Pan-Actin antibody is used as a control. Molecular weight is shown on the right side of the panel. (C) Relative mRNA and protein expression values of each enzyme in mouse ES cells and E8 embryos. ES mRNA and all protein data, N = 3; E8 mRNA, N = 5.

During differentiation from mouse ES cells to each tissue, knockdown of *AK* isoforms, *AK1*, *2* and *5*, in stem cells interfered with mitochondrial network formation and cardiac differentiation [33]. To further examine developmental regulation of adenine nucleotide metabolizing enzymes, we analyzed mRNA and protein expressions of Ak2, Ckmt1, Ckmt2, and Ndpk-d in two developmental stages of mouse ES cells and E8 embryos. As shown in Figure 2A, *Ak2* and *Ckmt1* mRNA were detected in mouse ES cells, whereas *Ckmt2* was not observed. In contrast, *Ak2* mRNA was similarly detected in E8 embryos, whereas both *Ckmt1* and *Ckmt2* mRNA were barely detected. *Ndpk-d* mRNA was weakly observed in both ES cells and E8 embryos. In addition, transcripts of both *Ak2* isoforms were detected in ES cells and E8 embryos (Figure S1C).

By western blot analysis, Ak2 protein was detected in both mouse ES cells and E8 embryos (Figure 2B), and Ndpk-d protein expression was slightly increased along development as shown in ES cells and E8 embryos. In contrast, both Ckmt1 mRNA and protein were detected in ES cells. These results demonstrated that expression of mitochondrial kinases are stage-specifically regulated during early embryonic stages (Figure 2C).

### AK2 Enzyme Activity in Mouse ES Cells, Embryos, and Adult Tissues

**Figure 3:**
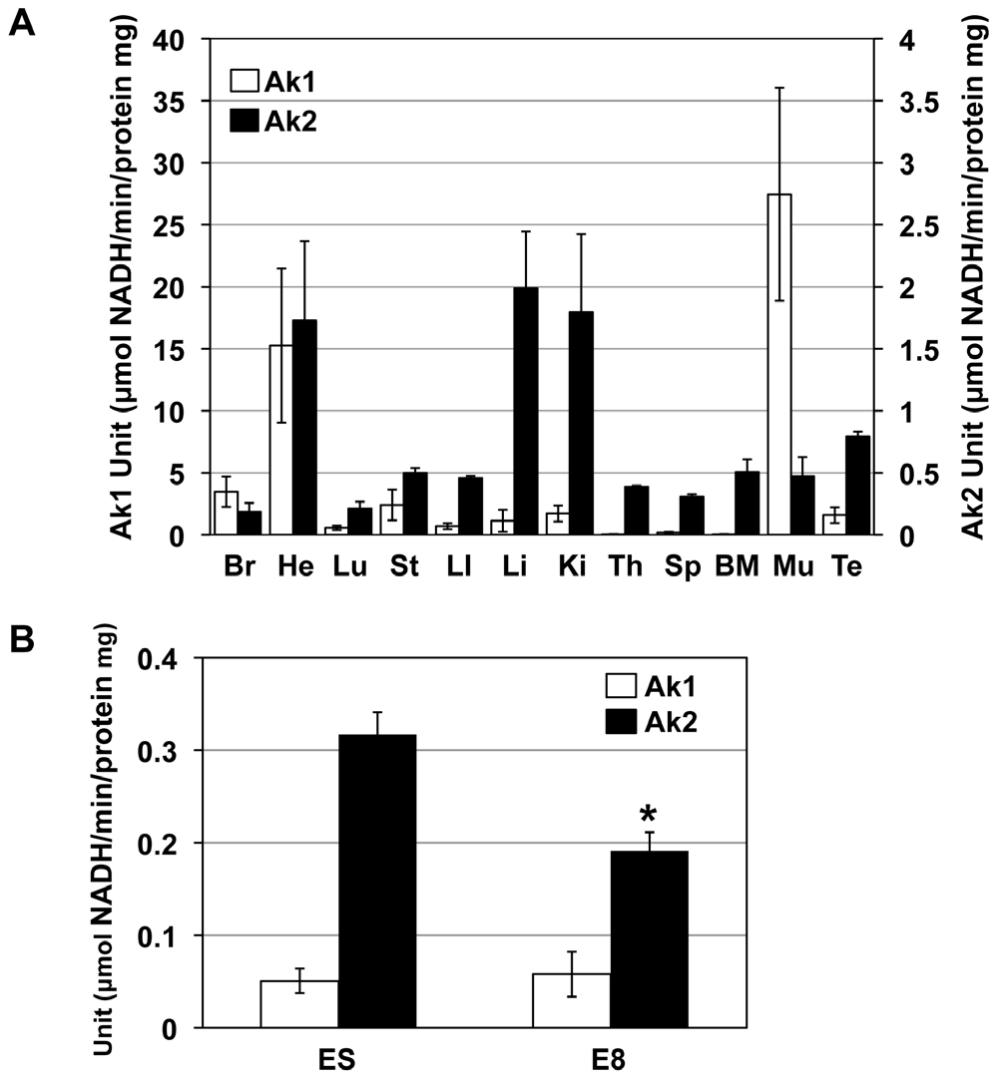
AK activity in mouse ES cells, E8 embryos, and 7-week-old adult mouse tissues. (A) AK1 and AK2 activities in adult mouse tissues; (B) AK1 and AK2 activities in mouse ES cells and E8 embryos. The activity of each AK was normalized to total protein contents. Open and closed bars indicate AK1 and AK2 activity, respectively. Br, brain; He, Heart; Lu, lung; St, stomach; LI, large intestine; Li, liver; Ki, kidney; Th, thymus; Sp, spleen; BM, bone marrow; Mu, skeletal muscle; and Te, testis. N = 3. *, p<0.05.

AK activities were measured in mouse ES cells, E8 embryos, and all 12 adult tissues. AK2 activities in adult tissues were roughly correlated with the levels of Ak2 protein expression, showing high in heart, liver, and kidney (Figure 3A). In contrast, AK1 activity was much higher in heart and skeletal muscle than in other tissues. However, AK2 activity without AK1 activity was uniquely detected in spleen, thymus, bone marrow, and liver. Moreover, relatively high AK2 activities were detected in mouse ES cells and embryos, whereas AK1 activity was very low in them (Figure 3B). Finally, AK2 activity in ES cells was higher compared with that in embryos, consistent with the level of Ak2 protein expression, suggesting that Ak2 may play a distinct role in IMS during early embryonic stages.

### Expression of mitochondrial kinases during HL-60 myelocytic differentiation

Exclusive expression of AK2 in bone marrow suggests a causal connection between AK2 deficiency and RD phenotypes. Bone marrow contains hematopoietic precursor cells and common myeloid progenitor (CMP) cells that differentiate into neutrophils and monocytes/macrophages [34], [35]. Therefore, mitochondrial kinases may be differentially regulated during terminal differentiation from CMP to neutrophil or monocyte/macrophage lineages. In order to examine the expression profile of each kinase, we used ATRA- and PMA-treated HL-60 cells as an *in vitro* model of terminal differentiation to granulocytes and monocytes/macrophages [23], [24].

**Figure 4:**
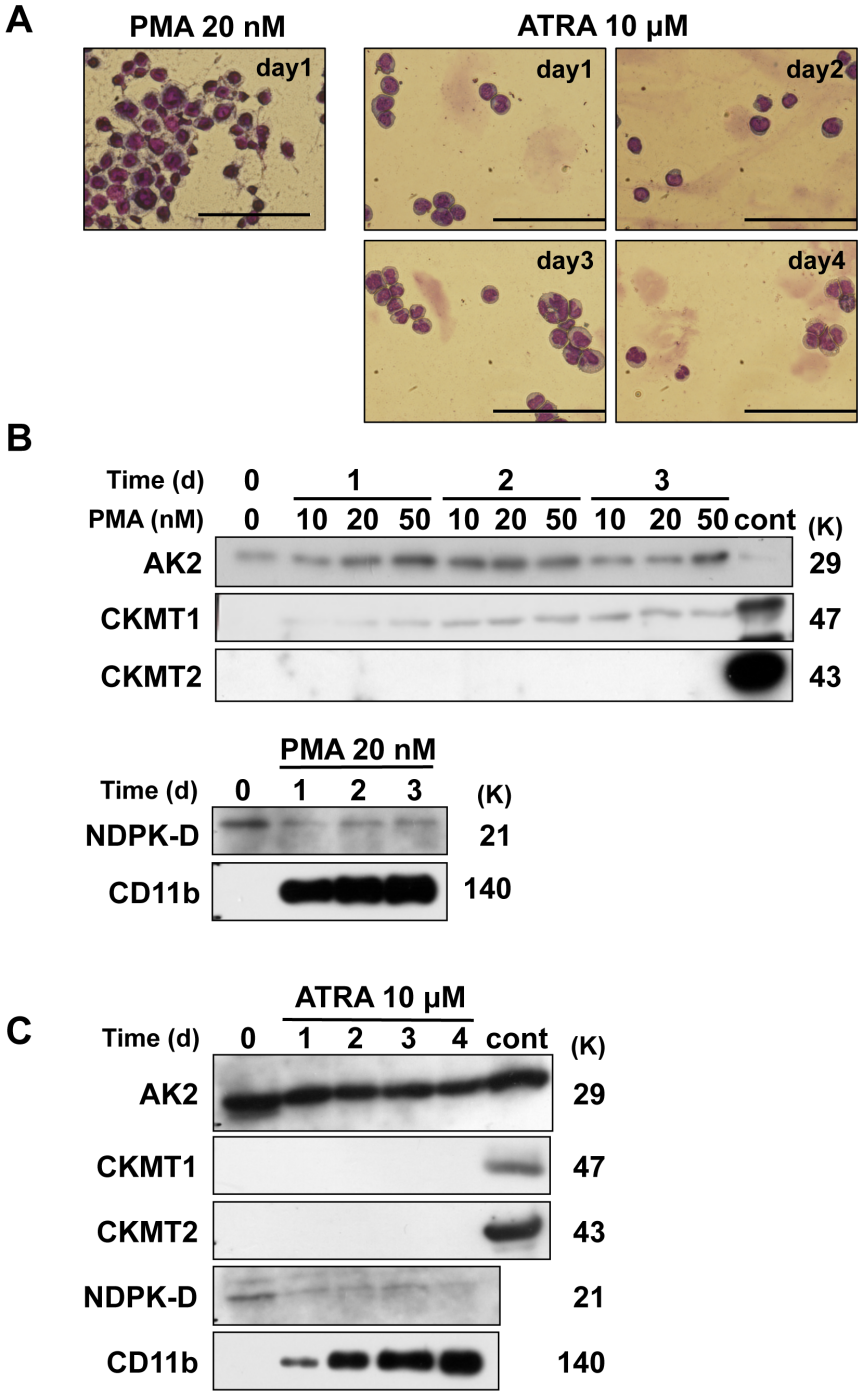
Regulation of mitochondrial kinase expression during HL-60 cell differentiation into macrophages or neutrophils. (A) Morphological confirmation of macrophage- and neutrophil differentiation of HL-60 cells. HL-60 cells were treated with 20 nM PMA for macrophage differentiation or 10 µM ATRA for neutrophil differentiation. PMA-treated HL-60 cells were stained with Wright-Giemsa, and ATRA-treated HL-60 cells were stained with Giemsa. Scale bar, 100 µm. (B, C) Analysis of enzyme expression during macrophage- (B) and neutrophil differentiation (C) of HL-60 cells. CD11b was used as a marker of myeloid differentiation. Cont indicates a positive control used as follows; skeletal muscle was used for AK2, CKMT1 and CKMT2 in B and for CKMT1 and CKMT2 in C, and kidney was used for AK2 in C.

Differentiation of HL-60 cells into monocytes/macrophages was induced after 1 day PMA treatment, as indicated by attachment of cells to culture dishes. The neutrophil differentiation of HL-60 cells was induced by 10 µM ATRA treatment for 4 days and was recognized by morphologically segmented nuclei (Figure 4A). After confirming morphology, we analyzed expression patterns of AK2, CKMT1, CKMT2, and NDPK-D during macrophage- and neutrophil differentiation. AK2 signals were increased in a dose-dependent manner during macrophage differentiation. CKMT1 expression, which was not detected on day 0, was weakly induced over the course of macrophage differentiation (Figure 4B), whereas CKMT2 remained undetectable. In contrast, constant AK2 expression was detected during neutrophil differentiation from days 0 to 4, whereas CKMT1 and CKMT2 remained undetectable throughout the time course (Figure 4C). In both differentiations, significant induction of the myeloid differentiation marker CD11b was confirmed in both macrophage- and neutrophil differentiation. Interestingly, NDPK-D signals were decreased during differentiation, suggesting that NDPK-D does not play an important role in adenine nucleotide metabolism in IMS during macrophage- and neutrophil differentiation. We further examined *AK2A* and *AK2B* mRNA expressions in human HL-60 cells during macrophage- and neutrophil differentiations. The similar levels of both *AK2* transcripts were detected in HL-60 cells during macrophage- and neutrophil differentiation, indicating that the alternative splicing of *AK2* isoforms is not affected by myeloid differentiation, at least, in our samples examined (Figure S1D).

### Effects of AK2 knockdown on macrophage- and neutrophil differentiation

**Figure 5:**
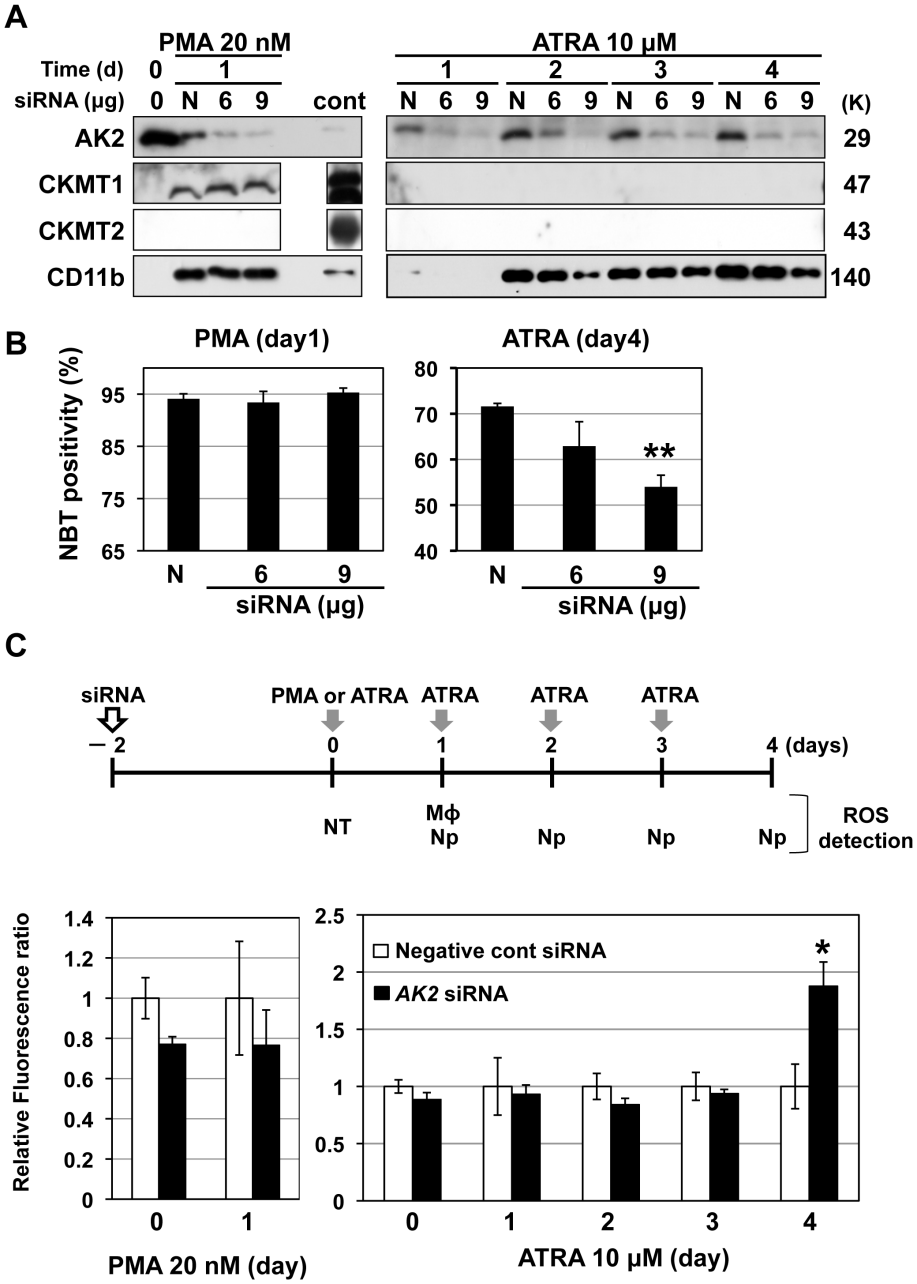
Effects of *AK2* knockdown on macrophage- and neutrophil differentiation of HL-60 cells. (A) Effects of *AK2* knockdown on macrophage- and neutrophil differentiation; N, control siRNA treatment; CD11b, differentiation marker; cont, mouse heart (AK2, CKMT1 and 2), human leukocyte (CD11b). (B) Rates of myeloid differentiation were assessed using NBT assays. Differentiated macrophage- and neutrophil HL-60 cells are NBT positive. N, control siRNA treatment; ** p<0.01. Experiments were performed in triplicate. (C) ROS measurement in HL-60 cells during macrophage- and neutrophil differentiations. Relative ratio of fluorescent intensity correlated to ROS production during myeloid differentiations was assessed at the each time point of the upper time course. Negative control siRNA treated samples, N = 3; *AK2* siRNA treated samples, N = 4. * p<0.05.

To further clarify the role of AK2 in macrophage- and neutrophil differentiation, we performed *AK2* knockdown experiments in HL-60 differentiation model (Figure 5A). CD11b expression in macrophage-differentiating HL-60 cells was impervious to *AK2* and control siRNAs. Functional maturity of differentiated myeloid cells was assessed according to the phagocytic reduction of the NBT dye. Furthermore, differentiation rates were assessed by counting NBT-positive cells with phagocytic and reducing activity. Interestingly, the number of NBT-positive cells was not affected by *AK2* knockdown (Figure 5B, left panel), indicating that AK2 does not play a critical role in the macrophage differentiation of HL-60 cells. In addition, *AK2* knockdown had no effect on CKMT1 expression (Figure 5A, upper panel).

On the other hand, CD11b expression was decreased by *AK2* knockdown in a dose-dependent manner during neutrophil differentiation (Figure 5A, lower panel). Furthermore, the NBT assay revealed significant reductions in neutrophil differentiation rates caused by *AK2* knockdown (Figure 5B, right panel).

In addition, we further examined ROS production and ATP levels in *AK2* knockdown during myelocytic differentiation, since enhanced ROS production was demonstrated in fibroblasts of individuals with RD [19]. We found that ROS level was specifically increased in *AK2*-knockdown HL-60 cells at day 4 of ATRA treatment compared with control, but not in PMA-treated HL-60 (Figure 5C), suggesting that neutrophil differentiation promotes ROS-induced damage under the condition of AK2 deficiency. On the other hand, ATP levels were reduced by *AK2*-knockdown treatment and maintained at the similar level during both macrophage- and neutrophil differentiation (Figure S2). These results indicated that AK2 among the mitochondrial kinases may play a major role in mitochondria during neutrophil differentiation.

### Discussion

Previous studies have demonstrated that AK2 deficiency impairs ontogenesis and cellular differentiation [17], [19]–[22], suggesting that ADP recycling by AK2 in IMS is essential for these processes. However, roles of the three other mitochondrial kinases such as CKMT1, CKMT2 and NDPK-D in the development of RD phenotypes remain unclear and still an open question. These kinases catalyze the formation of ADP, which functions as a substrate for ATP synthase [1], [2], [5], [9], [16], [36], [37]. To clarify the roles of AK2 and other mitochondrial kinases in hematopoietic differentiation, we determined the expression patterns and functions of them in both mouse tissues and cultured cells.

### Expression patterns of mitochondrial kinases

Expression profiles of Ak2, Ckmt1, Ckmt2 and Ndpk-d revealed tissue-specific and developmental stage-specific regulation, as shown in Figures 1 and 2, respectively. As previously demonstrated [14], high AK2 activities were detected in liver, kidney and heart tissues of mice (Figure 3). However, Ndpk-d protein levels did not change during development and were observed in all tissues, except bone marrow, suggesting that Ndpk-d may have housekeeping rather than developmental roles. In contrast, Ckmt1 and Ckmt2 were highly expressed in brain, heart, stomach, large intestine and skeletal muscle, thereby indicating that tissue-specific expression of mitochondrial enzymes individually or cooperatively contributes toward adenine nucleotide homeostasis. In this study, we found that the combinatory expression patterns of mitochondrial kinase are varied in each tissue. In particular, Ak2 is exclusively expressed in bone marrow, suggesting a unique role of AK2 in ADP recycling in IMS.

We also analyzed the developmental regulation of the mitochondrial kinases, Ak2, Ckmt1 and Ndpk-d, which were all expressed in mouse ES cells. Subsequently, Ak2 and Ndpk-d were dominantly expressed in E8 embryos, whereas AK2 activity was reduced by half in ES cells. Nevertheless, AK2 activity may not be critical in mammalian embryonic development as observed in *D. melanogaster* [17]. In fact, RD patients with *AK2* mutations were safely born [19], [20], possibly because of compensatory mechanism including glycolytic bioenergetics.

### Role of AK2 in hematopoietic differentiation

The expression analyses reveal high Ak2 expression in hematopoietic bone marrow tissue, suggesting an important role of AK2 in hematopoiesis. Accordingly, we characterized effects of *AK2* knockdown on myelocytic differentiation using human bipotent hematopoietic HL-60 cells as an *in vitro* model [23], [24].

Expression of mitochondrial kinases was uniquely observed during myelocytic differentiation, with gradually increased AK2 and CKMT1 expression during macrophage differentiation (Figure 4B). In contrast, AK2 was dominantly and constantly expressed compared with other enzymes during neutrophil differentiation (Figure 4C). According to these findings and previous reports [19], [20], we examined whether AK2 deficiency disturbs neutrophil differentiation leading to neutropenia. In these experiments, we found that *AK2* knockdown particularly inhibited neutrophil differentiation, but not macrophage, (Figure 5), indicating a cell-type specific role of AK2 during myeloid differentiation.

**Figure 6:**
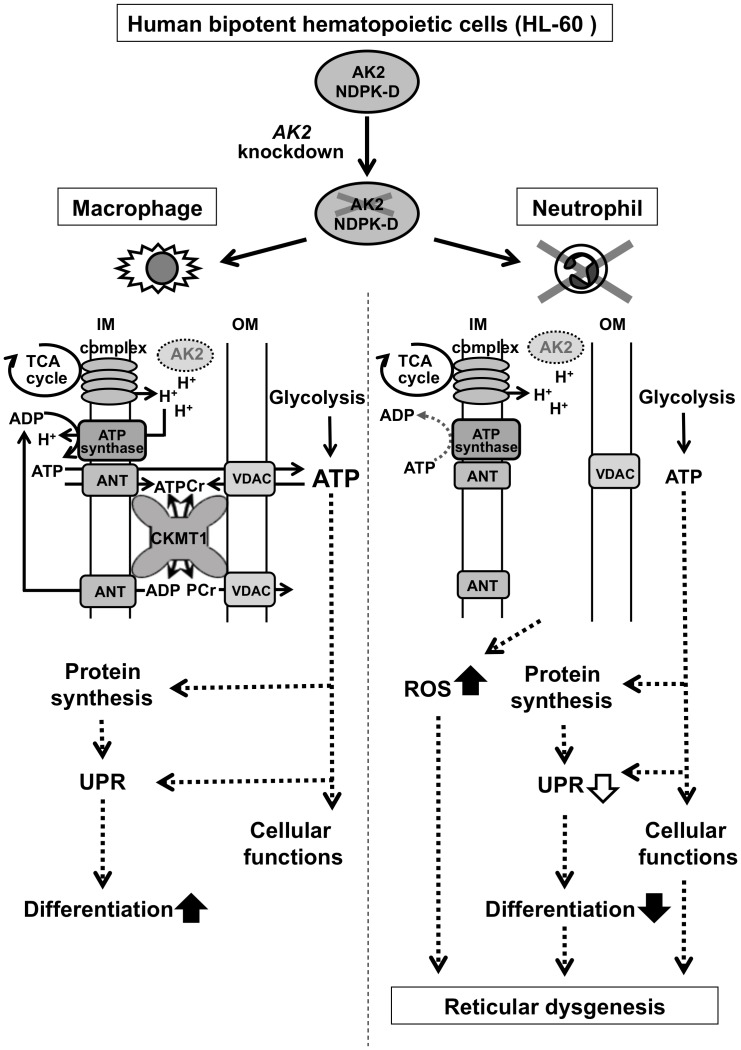
Working hypothesis for the role of AK2 during hematopoietic differentiation of bipotent HL-60 progenitor cells. Human *AK2*-deficient hematopoietic progenitor cells can differentiate into macrophages but not into neutrophils (upper panel). During differentiation into macrophages (left panel), CKMT1 may interact with mitochondrial ATP synthase, ANT, and voltage-dependent anion channel (VDAC). This interaction recycles ATP-ADP in IMS without AK2. ATP from CKMT1-mediated ADP recycling could be used for cellular function including UPR to decrease ER stress induced by *de novo* neosynthesized proteins and to support macrophage differentiation. During neutrophil differentiation (right panel), *AK2*-deficient hematopoietic progenitor cells could not fully maintain mitochondrial adenine nucleotide homeostasis. The subsequent ER and oxidative stresses may impair differentiation and cellular functions. Disturbed adenine nucleotide metabolism in IMS may lead to ER stress and abnormal ROS production as shown in patients with RD and our *AK2*- deficient experimental models resulting in either neutropenia or impairment of neutrophil differentiation. IM and OM indicate mitochondrial inner and outer membranes, respectively. Open arrows and dotted-lines indicate the possible regulations, filled allows are the confirmed findings in this study.

Based on these findings, we propose the following working hypothesis for the molecular basis of RD (Figure 6). Loss of mitochondrial adenine nucleotide metabolizing enzymes such as AK2, CKMT1 and CKMT2 in IMS, may result in impairment of ADP recycling, which weakens mitochondrial ATP production. Subsequently, mitochondrial dysfunction may trigger (1) uncontrolled leakage of electrons from the electron transfer chain and ATP deficit, and (2) impairment of unfolded protein response (UPR) to the increasing demand for protein production during somatic cell differentiation. Finally, reactive oxygen species and ER stress may be increased [38]–[40]. In agreement with this model, it was recently reported that AK2 is important for UPR activity in ER during adipocyte and B cell differentiation [21]. It is not unclear how *AK2* knockdown affects neutrophil differentiation. As one of possible mechanisms, Burkart *et al* reported that AK2 deficiency blocks adipocyte differentiation directly through UPR and/or ER stress [21]. Therefore, we also examined the levels of IRE1 and sXBP1 expression and found that they were decreased by *AK2* knockdown in a HL-60 model of neutrophil differentiation (data not shown), suggesting that AK2 deficiency might be linked with ER stress during neutrophil differentiation. However, further investigation is required for better understanding the relationship between ADP recycling across IMS and ER stress including UPR.

In addition, under physiological conditions, ROS production in mitochondria plays important roles in cellular differentiation and cell signaling [41], [42]. However, excessive ROS production under pathological conditions such as ischemia and mitochondrial membrane depolarization is detrimental to cellular function [43]. In our study, *AK2*-knockdown treatment specifically enhanced ROS production in ATRA-induced neutrophil differentiated HL-60 cells (Figure 5C). Moreover, in *Ant1*-deficient mouse as a disruption model of oxidative phosphorylation, it was reported that blocking of ATP and ADP exchange across the mitochondrial inner membrane resulted in high levels of ROS production [44]. When matrix ADP is not enough supplied, ATP synthase is no more able to synthesize ATP because of substrate deficiency, causing uncoupling with respiration in mitochondria. Thereby, inhibition of the ATP synthase by impairment of ADP recycling may affect the electrochemical gradient, ROS production from Complexes I and III of the mitochondrial electron transfer chain [45], and impairment of mitochondrial membrane potential [46]. However, activation of oxidative phosphorylation by recycled ADP would increase respiratory rates and decrease mitochondrial membrane potential and ROS production [46]. In agreement with this scenario, creatine limitation enhanced ROS production in rat brains and embryonic cortical neurons, wherein only Ckmt1 is expressed as an ADP recycler of IMS, indicating that ADP recycling by Ckmt1 prevents intracellular ROS generation [40]. In addition, it was demonstrated that yeast mitochondrial AK located close to ANT functions to supply ADP to ATP synthase in matrix, whereas disruption of mitochondrial AK would prevent ANT from ATP export in exchange for ADP [47]. These findings suggest that defects of mitochondrial adenine nucleotide metabolizing kinases may lead to proton accumulation, hyperpolarization, and excessive ROS production resulting from impairment of ATP-ADP recycling. Therefore, AK2 may play the critical roles to maintain mitochondrial ADP and ATP levels, control ROS production, and regulate cell-fate. AK2 has been reported to be released during apoptosis [48] and to form a complex with FADD and caspase-10 and involved the decision of cell death or alive [49]. Further detailed investigation is required for understanding the relationship among ATP synthesis, H^+^ generation, and ROS production including apoptosis.

In conclusion, we found that mitochondrial adenine nucleotide metabolizing enzymes Ak2, Ckmt1, Ckmt2, and Ndpk-d were differentially regulated according to tissue types and differentiation stages. Exclusive expression of AK2 during neutrophil differentiation may link AK2 deficiency and impairment of neutrophil differentiation as a possible causative mechanism of RD. Further studies are required to confirm the regulatory role of AK2 in physiological hematopoiesis and to develop corresponding therapeutic strategies for RD.

## Supporting Information

Figure S1 ***AK2*** **isoform-expressions in adult mouse tissues, ES cells, E8 embryos and HL-60 cells.** (A) Primer designs for human *AK2* isoforms and mouse *Ak2* isoforms. Arrows indicate isoform-specific primers of each species. The following primers were used; *Ak2AB* forward 5′-CTGTTGGAGTGAAGCTTTGG-3′, *Ak2A* reverse 5′-CTAACCATCACCACCCACTC-3′, *Ak2B* reverse 5′-GCACCTAAGAGCAGGGATCC-3′, *AK2AB* forward 5′-GTGGCAGTGAGAGACTTCGG-3′, *AK2A* reverse 5′-CCTATCATTCCCACCCATTG-3′, *AK2B* reverse 5′-GCACCTAAGAGCAGGGATCA-3′. (B) Tissue-specific expression of *Ak2A* and *Ak2B* mRNA in adult mouse tissues. Br, brain; He, Heart; Lu, lung; St, stomach; LI, large intestine; Li, liver; Ki, kidney; Th, thymus; Sp, spleen; BM, bone marrow; Mu, skeletal muscle; Te, testis. (C) *Ak2A* and *Ak2B* mRNA expressions in mouse ES cells and E8 embryos. ES, mouse ES cells; E8, mouse E8 embryos. (D) *AK2A* and *AK2B* mRNA expressions during 3 sets of macrophage differentiation by PMA treatment and 2 sets of neutrophil differentiation by ATRA treatment in human HL-60 cells. *18S rRNA* is presented as a control.
(TIF)

Figure S2 **ATP measurement in HL-60 cells during macrophage- and neutrophil differentiations.** ATP amount was measured using CellTiter-Glo Luminescent Cell Viability Assay (Promega) according to the manufacturer's instruction. Data were shown by relative light units (RLU) of luciferase activity during myeloid differentiation as shown in ROS assay (Figure 5C). Open bar; control siRNA treatment (N = 3), closed bar; *AK2* siRNA treatment (N = 4), * p<0.05, ** p<0.01.
(TIF)
