# Correction: Differential Expression of Adenine Nucleotide Converting Enzymes in Mitochondrial Intermembrane Space: A Potential Role of Adenylate Kinase Isozyme 2 in Neutrophil Differentiation

**DOI:** 10.1371/journal.pone.0099247

**Published:** 2014-05-28

**Authors:** The PLOS ONE Staff

An incorrect grant number is in the funding statement. The correct funding statement is as follows: This work was partly supported by a Grant-in-Aid for Young Scientists (B) 25750042 from the Ministry of Education, Science, Sports and Culture, a grant for the special scientific research program aimed at the improvement of QOL (Quality of Life) via oral function in collaboration with universities from the Ministry of Education, Culture, Sports, Science, and Technology, and a research grant supported by Kojinkai Foundation, and the President discretion research budget of the University of Tokushima. No additional external funding received for this study. The funders had no role in the study design, data collection and analysis, decision to publish, or preparation of the manuscript.
