# Supplementary figures and images for: Differential Expression of Adenine Nucleotide Converting Enzymes in Mitochondrial Intermembrane Space: A Potential Role of Adenylate Kinase Isozyme 2 in Neutrophil Differentiation
Source: PLoS One. 2014 Feb 25;9(2):e89916. doi: 10.1371/journal.pone.0089916 (PMC3934953; doi:10.1371/journal.pone.0089916)

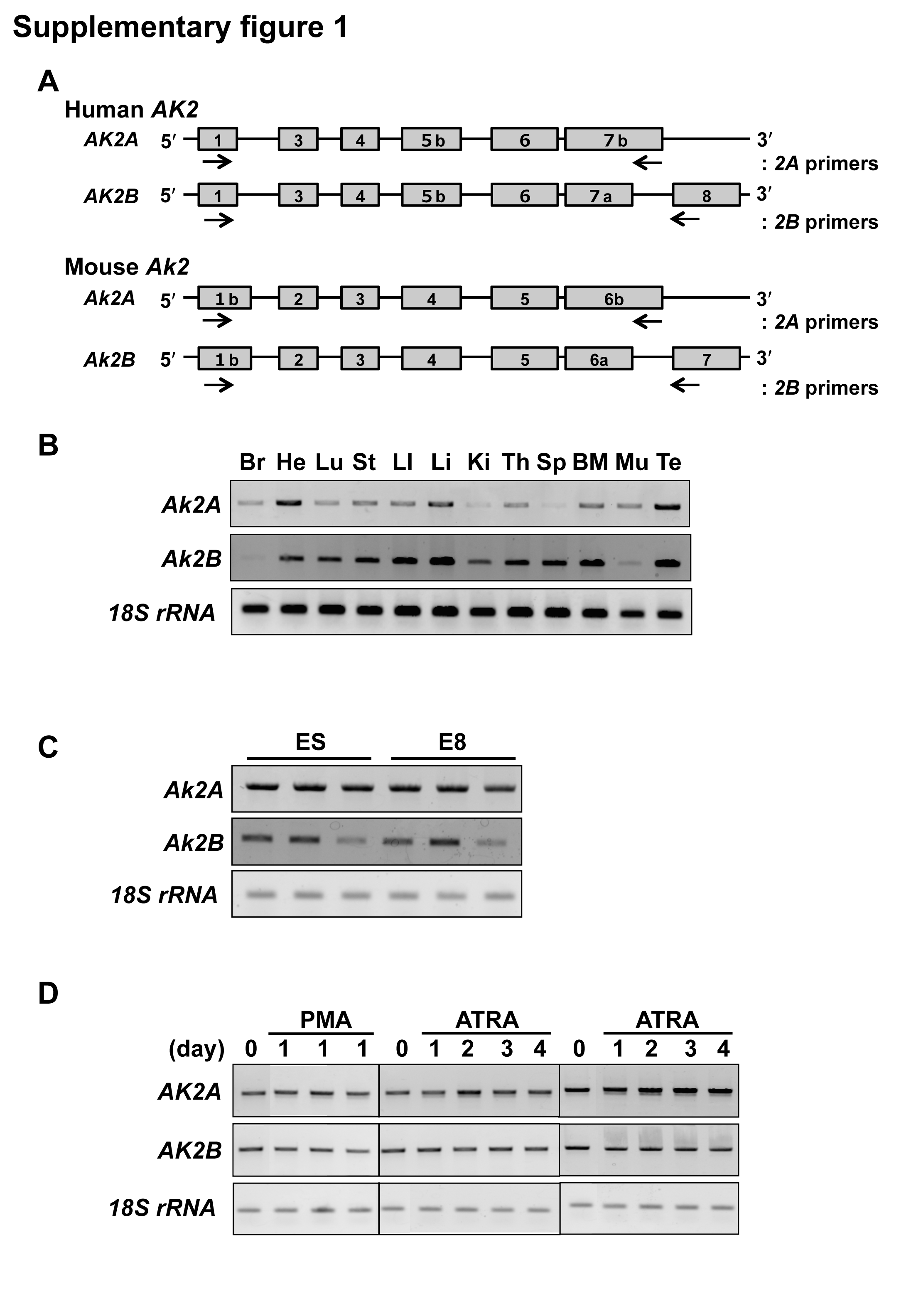

Supplement: Figure S1 — AK2 isoform-expressions in adult mouse tissues, ES cells, E8 embryos and HL-60 cells. (A) Primer designs for human AK2 isoforms and mouse Ak2 isoforms. Arrows indicate isoform-specific primers of each species. The following primers were used; Ak2AB forward 5′-CTGTTGGAGTGAAGCTTTGG-3′, Ak2A reverse 5′-CTAACCATCACCACCCACTC-3′, Ak2B reverse 5′-GCACCTAAGAGCAGGGATCC-3′, AK2AB forward 5′-GTGGCAGTGAGAGACTTCGG-3′, AK2A reverse 5′-CCTATCATTCCCACCCATTG-3′, AK2B reverse 5′-GCACCTAAGAGCAGGGATCA-3′. (B) Tissue-specific expression of Ak2A and Ak2B mRNA in adult mouse tissues. Br, brain; He, Heart; Lu, lung; St, stomach; LI, large intestine; Li, liver; Ki, kidney; Th, thymus; Sp, spleen; BM, bone marrow; Mu, skeletal muscle; Te, testis. (C) Ak2A and Ak2B mRNA expressions in mouse ES cells and E8 embryos. ES, mouse ES cells; E8, mouse E8 embryos. (D) AK2A and AK2B mRNA expressions during 3 sets of macrophage differentiation by PMA treatment and 2 sets of neutrophil differentiation by ATRA treatment in human HL-60 cells. 18S rRNA is presented as a control. (TIF) [file pone.0089916.s001.tif]

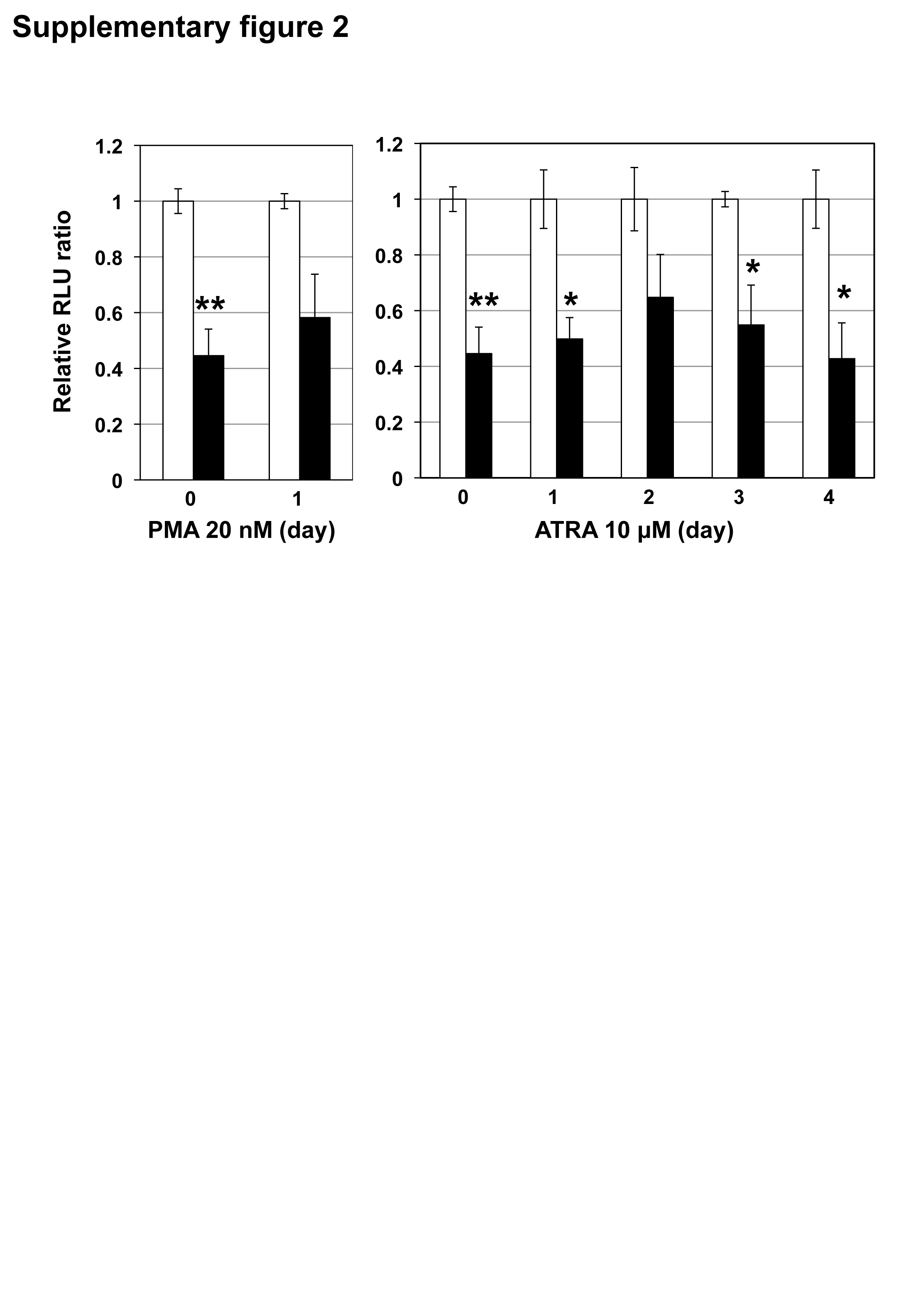

Supplement: Figure S2 — ATP measurement in HL-60 cells during macrophage- and neutrophil differentiations. ATP amount was measured using CellTiter-Glo Luminescent Cell Viability Assay (Promega) according to the manufacturer's instruction. Data were shown by relative light units (RLU) of luciferase activity during myeloid differentiation as shown in ROS assay (Figure 5C). Open bar; control siRNA treatment (N = 3), closed bar; AK2 siRNA treatment (N = 4), * p<0.05, ** p<0.01. (TIF) [file pone.0089916.s002.tif]
